# The Association between Individual and Combined Components of Metabolic Syndrome and Chronic Kidney Disease among African Americans: The Jackson Heart Study

**DOI:** 10.1371/journal.pone.0101610

**Published:** 2014-07-03

**Authors:** Vincent L. Mendy, Mario J. Azevedo, Daniel F. Sarpong, Sylvia E. Rosas, Olugbemiga T. Ekundayo, Jung Hye Sung, Azad R. Bhuiyan, Brenda C. Jenkins, Clifton Addison

**Affiliations:** 1 Department of Epidemiology and Biostatistics, School of Health Sciences, College of Public Service, Jackson State University, Jackson, Mississippi, United States of America; 2 RTRN Data Coordinating Center, Jackson State University, Jackson, Mississippi, United States of America; 3 Joslin Diabetes Center, Kidney and Hypertension Section, Harvard Medical School, Boston, Massachusetts, United States of America; 4 Jackson Heart Study, Jackson State University, Jackson, Mississippi, United States of America; Universtiy of Maryland Schoool of Medicine, United States of America

## Abstract

**Introduction:**

Approximately 26.3 million people in the United States have chronic kidney disease and many more are at risk of developing the condition. The association between specific metabolic syndrome components and chronic kidney disease in African American individuals is uncertain.

**Methods:**

Baseline data from 4,933 participants of the Jackson Heart Study were analyzed. Logistic regression models were used to estimate the odds and 95% confidence intervals of chronic kidney disease associated with individual components, metabolic syndrome, the number of components, and specific combinations of metabolic syndrome components.

**Results:**

Metabolic syndrome was common with a prevalence of 42.0%. Chronic kidney disease was present in 19.4% of participants. The prevalence of metabolic components was high: elevated blood pressure (71.8%), abdominal obesity (65.8%), low fasting high density lipoprotein cholesterol (37.3%), elevated fasting glucose (32.2%) and elevated triglycerides (16.2%). Elevated blood pressure, triglycerides, fasting blood glucose, and abdominal obesity were significantly associated with increased odds of chronic kidney disease. Participants with metabolic syndrome had a 2.22-fold (adjusted odds ratio (AOR) 2.22; 95% CI, 1.78–2.78) increase in the odds of chronic kidney disease compared to participants without metabolic syndrome. The combination of elevated fasting glucose, elevated triglycerides, and abdominal obesity was associated with the highest odds for chronic kidney disease (AOR 25.11; 95% CI, 6.94–90.90).

**Conclusion:**

Metabolic syndrome as well as individual or combinations of metabolic syndrome components are independently associated with chronic kidney disease in African American adults.

## Introduction

Recent studies estimate that approximately 26.3 million people in the United States have chronic kidney disease (CKD) [1]. There is a gap in awareness of the condition among high risk adults [2], particularly minority populations [3]. There are racial differences in CKD [4]. African American (AA) adults develop CKD at higher rates than whites, namely during middle age, and experience a disproportionately greater burden of Stage 5 CKD [5]. Compared to other racial/ethnic groups, CKD tends to progress to end stage renal disease (ESRD) more frequently in AAs [6], [7]. The drastic increase in the burden of CKD with regards to human suffering and economic cost and its link to CVD makes it an imperative public health issue in the 21^st^ century [8], [9].

Metabolic syndrome (MetS) is the clustering of risk factors for cardiovascular disease (CVD) and Type 2 diabetes mellitus [10]. Components of MetS include abdominal obesity, elevated blood pressure, plasma triglycerides, fasting blood glucose and low fasting high-density lipoprotein (HDL) cholesterol [11]. An association between MetS and CKD has been found in population-based cohorts and cross-sectional studies [12]–[15]. Ford et al. (2004) [16] found that the unadjusted prevalence of MetS increased over time among U.S. adult cohorts, from 23.1% in the National Health and Nutrition Examination Survey (NHANES) III (1988–1994) to 26.7% in NHANES 1999–2000, and 34% in NHANES 2003–2006. Using data from the Jackson Heart Study (JHS) [17], Taylor and colleagues found that 43.3% of women and 32.7% of men had MetS [18]. The prevalence of CKD is higher among Mississippians than among the overall U.S. population [19] and MetS prevalence in the JHS is among the highest globally [18], [20]. Few studies have examined the association between MetS and CKD among minority populations. Here we report the associations between the presence of MetS, individual MetS components and CKD. Additionally, we sought to determine the association between number of MetS components and CKD risk, as well as explored combinations of MetS components and CKD risk among AA adults in JHS.

## Methods

### Study design and study population

The JHS study is designed to monitor etiology and progression of CVD prospectively in AAs in Mississippi. JHS population includes non-institutionalized AAs age 21 years and older in the Jackson, Mississippi Metropolitan Statistical Area (MSA) counties (Hinds, Madison, and Rankin) [21]. The final JHS cohort included 5,301 participants from the baseline (Visit 1) examination period of September 2000 to March 2004. Detailed descriptions of the study’s design, procedures, recruitment and sample size have been published elsewhere [21], [22]. This study was approved by the institutional review boards of Jackson State University, Tougaloo College and the University of Mississippi Medical Center. All of the participants gave written informed consent.

The baseline examination included a home visit, self-administered questionnaires and a clinic visit. During clinical examination participants were asked about their medication use in the past two weeks. Participants were asked to fast overnight before the clinical examination, venous blood was obtained and sitting blood pressure recorded using standard Hawksley random-zero instruments according to established blood pressure measurement guidelines [3], [23]. Urine samples were obtained according to the National Committee for Clinical Laboratory Standards [24] and sociodemographic information was obtained thorough self-administered questionnaires.

### Study variables

CKD was defined as an estimated glomerular filtration rate (eGFR) of <60 mL/min/1.73 m^2^, or the presence of albuminuria, or being on dialysis according to the National Kidney Foundation (NKF) guidelines [25]; coded as “present” and “absent”. Albuminuria was defined as urine albumin to urine creatinine ratio >30 mg/g based on spot or 24-hour urine values. eGFR was calculated based on the 4-variable Modification of Diet in Renal Disease (MDRD) Study equation [GFR = 186.0 × (serum creatinine)^−1.154^×(age)^−0.203^×(0.742 if female) × (1.210 if African American)].

MetS was defined according to the Adult Treatment Panel Third Report (ATP III) [9]; coded as “yes” or “no.” MetS status was considered positive if the study participant had three or more of the following: 1) abdominal obesity, defined by a waist circumference ≥102 cm in men and ≥88 cm in women; 2) elevated blood pressure, defined as a systolic blood pressure of 130 mm Hg or greater and/or a diastolic blood pressure of 85 mm Hg or greater, physician diagnosis, and/or use of antihypertensive drugs; 3) elevated plasma triglycerides (≥150 mg/dL) or treated dyslipidemia; 4) low fasting HDL cholesterol (men <40 mg/dL and women <50 mg/dL; and 5) elevated fasting glucose (≥100 mg/dL) or use of anti-diabetic medication.

### Statistical analysis

Baseline characteristics were described using means and standard deviations for continuous variables and counts and percentages for categorical variables. Comparisons of baseline characteristics by CKD status (present/absent) were assessed using t-tests and chi-square tests for continuous and categorical variables, respectively. MetS status (yes/no), the presence of individual MetS components, and the number (0 to 5) of MetS components were determined. Multiple logistic regression models and 95% confidence intervals were used to estimate the odds of having CKD that are associated with MetS status, individual MetS components, and the number of MetS components when age, gender, education, income, active living index (physical activity during leisure time excluding sports), body mass index (BMI), current cigarette smoking, and the use of non-steroidal anti-inflammatory drugs (NSAIDs) are held constant. Participants with one, two, three, four, or five MetS components were compared to those having no MetS components and participants with MetS were compared to those with fewer than three components of MetS. The odds ratios of having CKD that are associated with ten combinations of three components and five combinations of four components of MetS were assessed and compared with the odd ratios for participants with zero components to ascertain the combinations with the highest odds, controlling for covariates. SAS version 9.3 (SAS Institute, Inc., Cary, North Carolina) was used to perform all statistical analyses; significance levels were determined at p<0.05, two tailed.

## Results

### Characteristics of study participants at baseline

In the current analysis, 368 JHS participants were excluded because of missing data on CKD. The analytical sample included 4,933 participants from baseline (Exam 1, 2000–2004). The mean age of participants was 54.9 years (±12.8; range, 21–95), and 63.9% were female. A more detailed description of the socio-demographic characteristics of the sample participants is presented in Table 1.

**Table 1:** Sociodemographic Characteristics of Jackson Heart Study Participants at Baseline, 2000–2004.

Characteristic	N = 4,933	
	n	%
Age in years [mean (SD)]	54.9 (12.8)	
Female gender	3,154	63.9
Family income		
Low^a^	642	15.3
Lower-middle^b^	1,053	25.1
Upper-middle^c^	1,243	29.7
Affluent^d^	1,250	29.9
Education level		
Less than high school	891	18.1
High school/GED	983	20.0
Vocational school or some college/Associate’s degree	1,428	29.1
Bachelor’s degree or higher	1,612	32.8
Marital status		
Married	2,692	54.8
Never been married	610	12.4
Separated	204	4.2
Divorced	744	15.1
Widowed	666	13.6

SD: standard deviation; GED, general educational development.

a Low = ≤ $11,999, ^b^Lower-middle = $12,000–24,999, ^c^Upper-middle = $25,000–49,999, ^d^Affluent = ≥ $50,000.

### MetS, individual MetS components, and CKD

Among study participants, 71.8% had elevated blood pressure; 65.8% were defined as having abdominal obesity; 37.3% had low fasting HDL cholesterol; 32.2% had elevated fasting glucose; and 16.2% had elevated triglycerides. MetS was common with a prevalence of 42.0%; the prevalence of CKD was 19.4%.

### Comparison of study participants by CKD status

Compared to those without CKD, participants with CKD were older (mean age 61.1 vs. 52.7 years, p<0.001) and the proportion of women with CKD was higher compared with those without (67.7% vs. 62.5%, p = 0.02). The percent of participants who were affluent, married, and reported a high school education or higher, excellent or good health status, and drinking alcohol in the past 12 months was significantly lower among those without CKD than those with CKD (p<0.001). Study participants with CKD had significantly higher mean BMI, waist circumference, systolic blood pressure, fasting glucose, fasting total cholesterol, fasting triglycerides (p<0.001), and fasting low density lipoprotein (LDL) cholesterol (p = 0.01) than those without CKD (Table 2).

**Table 2:** Comparative Analysis of Characteristics of Jackson Heart Study Participants by Chronic Kidney Disease Status at Baseline, 2000–2004.

Characteristics	CKD		p-value^b^
	Present	Absent	
	(n = 628)	(n = 2,602)	
Age (years)	61.05±12.60	52.68±12.66	<.001
Gender (% women)	425 (67.68)	1,625 (62.45)	0.02
Education (% ≥ high school)	453 (72.60)	2,259 (86.92)	<0.001
Income (% affluent)	109 (21.08)	739 (33.82)	<0.001
Married status (%)	294 (47.04)	1,477 (56.83)	<0.001
Insured (%)	548 (88.10)	2,259 (87.09)	0.49
Health status (% excellent/good)	340 (54.40)	1,936 (74.43)	<0.001
Utilized preventive care (%)	488 (78.21)	1,838 (70.86)	<0.001
Alcohol drinking in past 12 months (%)	205 (32.75)	1,216 (46.86)	<0.001
Current smoker (%)	68 (22.12)	289 (11.17)	0.02
Body mass index (kg/m^2^)	33.26±7.64	31.43±6.91	<0.001
Active living index^a^	1.92±0.80	2.11±0.79	<0.001
Type 2 diabetes (% yes)	244 (39.10)	334 (12.86)	<0.001
Waist circumference (cm)	105.5±16.67	99.41±15.82	<0.001
Systolic blood pressure (mm Hg)	135.4±21.69	124.3±16.69	<0.001
Diastolic blood pressure (mm Hg)	79.05±12.09	79.10±9.97	0.93
Hypertension (% yes)	549 (87.70)	1,473 (56.70)	<0.001
Fasting blood glucose (mg/dL)	111.10±45.13	95.76±26.15	<0.001
Fasting total cholesterol (mg/dL)	205.2±45.02	197.2±38.68	<0.001
Fasting low HDL cholesterol (mg/dL)	50.68±14.89	50.91±13.96	0.72
Fasting LDL cholesterol (mg/dL)	130.7±40.38	126.0±35.65	0.01
Hypercholesterolemia (% yes)	269 (46.78)	764 (29.90)	<0.001
Fasting triglycerides (mg/dL)	122.4±74.74	103.0±79.26	<0.001
Hypertriglyceridemia (%)	64 (12.17)	165 (6.88)	<0.001
NSAIDs use (% yes)	363 (57.80)	1,045 (40.16)	<0.001

CKD, chronic kidney disease; HDL, high-density lipoprotein; LDL, low-density lipoprotein; eGFR, estimated glomerural filtration rate; SD, standard deviation; NSAIDs, non-steroidal anti-inflammatory drugs.

Values are expressed as mean ±SD for continuous variables and percentages for categorical variables.

a Active living index, defined as physical activity during leisure time excluding sports.

b Between-group comparisons: t test for continuous variables and chi-square for categorical variables.

### Association between individual components of MetS and CKD

The unadjusted and multivariable-adjusted odds ratios of having CKD for each individual component of MetS are presented in Table 3. The adjusted multivariable model shows that the presence of abdominal obesity, elevated triglycerides, elevated blood pressure, and elevated fasting glucose were significantly associated with an increased odds of CKD.

**Table 3:** Unadjusted and Multivariable-adjusted Odds of Having Chronic Kidney Disease for each Individual Component, Number of Components of Metabolic Syndrome and Metabolic Syndrome among Jackson Heart Study Participants at Baseline, 2000–2004.

Variable	Odds ratio of chronic kidney disease (95% CI)					
	UnadjustedOR (95% CI)	p-value^b^	Age-gender- adjustedOR (95% CI)	p-value^b^	Multivariable adjusted^a^OR (95% CI)	p-value^b^
Abdominal obesity	2.17 (1.77–2.67)	<0.001	2.01 (1.61–2.51)	<0.001	1.41 (1.06–1.89)	0.020
Elevated triglycerides	2.11 (1.68–2.65)	<0.001	1.88 (1.48–2.39)	<0.001	1.74 (1.33–2.28)	<0.001
Low HDL cholesterol	1.19 (0.99–1.43)	0.070	1.31 (1.08–1.59)	0.007	1.20 (0.96–1.50)	0.110
Elevated blood pressure	6.06 (4.45–8.26)	<0.001	4.03 (2.93–5.55)	<0.001	4.41 (2.99–6.49)	<0.001
Elevated fasting glucose	3.22 (2.69–3.85)	<0.001	2.41 (1.99–2.90)	<0.001	1.99 (1.60–2.47)	<0.001
0 components	Reference		Reference		Reference	
1 component	2.72 (1.48–4.97)	0.001	1.96 (1.06–3.62)	0.03	2.57 (1.20–5.51)	0.020
2 components	4.40 (2.46–7.88)	<0.001	3.03 (1.68–5.47)	<0.001	3.64 (1.72–7.72)	0.001
3 components	9.27 (5.22–16.47)	<0.001	5.64 (3.14–10.14)	<0.001	5.85 (2.75–12.46)	<0.001
4 components	11.23 (6.18–20.43)	<0.001	6.74 (3.66–12.40)	<0.001	6.82 (3.12–14.89)	<0.001
5 components	14.70 (7.43–29.08)	<0.001	8.59 (4.28–17.22)	<0.001	9.89 (4.17–23.49)	<0.001
Metabolic syndrome^c^	3.31 (2.76–3.97)	<0.001	2.68 (2.22–3.24)	<0.001	2.22 (1.78–2.78)	<0.001

OR, odds ratio; CI, confidence interval; HDL, high density lipoprotein.

a Adjusted for age, gender, education, income, active living index, body mass index (BMI), cigarette smoking, and non-steroidal anti-inflammatory drugs (NSAIDs).

b P-values calculated using logistic regression.

c Compared to those with <3 components of metabolic syndrome.

### Associations between MetS, the number of MetS components and CKD

**Figure 1:**
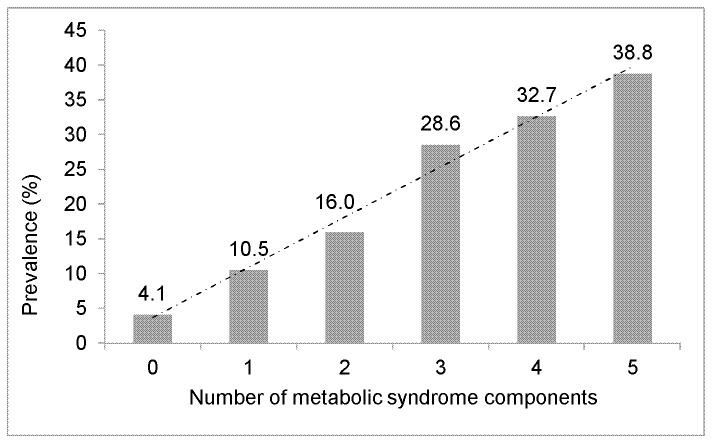
Prevalence of Chronic Kidney Disease by Number of Metabolic Syndrome Components among African American Adults, Jackson Heart Study, 2000–2004. Linear trend, *P*<0.001.

Without adjustment for covariates, participants with MetS had 3.3-fold higher odds of having CKD than those without MetS. Age- and gender-adjusted odds ratios indicate that participants with MetS had 2.68-fold higher odds of having CKD than those without MetS (p<0.001) (Table 3). Multivariable-adjusted odds ratios show that participants with MetS had 2.22-fold higher odds of having CKD than those without MetS (p<0.001). Figure 1 shows the prevalence of CKD by the number of MetS components present; the figure reveals a significant linear trend between the prevalence of CKD and the number of MetS components present.

### Association between combinations of MetS components and CKD

Table 4 presents the odds ratios of having CKD for each of the fifteen specific combinations of MetS components–ten combinations of three components and five combinations of four components. In multivariable analyses of the ten combinations of three MetS components, participants with the following three combinations had the highest odds of having CKD: 1) elevated fasting glucose, abdominal obesity, and elevated triglycerides (OR 25.11; 95% CI, 6.94–90.90); 2) elevated blood pressure, elevated triglycerides, and elevated fasting glucose (OR 14.98; 95% CI, 4.59–48.92); 3) low HDL cholesterol, elevated triglycerides, and elevated fasting glucose (OR 13.92; 95% CI, 3.40–56.98); and 4) elevated blood pressure, abdominal obesity, and elevated fasting glucose (OR 13.26; 95% CI, 5.21–33.72). Multivariable analyses of the five combinations of four MetS components show participants with the following four combinations had the highest odds of having CKD: 1) elevated blood pressure, abdominal obesity, elevated triglycerides, and elevated fasting glucose (OR 27.06; 95% CI, 7.21–101.60); and 2) abdominal obesity, low HDL cholesterol, elevated triglycerides, and elevated fasting glucose (OR 18.86; 95% CI, 3.89–91.73).

**Table 4:** Unadjusted and Multivariable-adjusted Odds of Having Chronic Kidney Disease for Specific Combinations of among Jackson Heart Study Participants at Baseline, 2000–2004.

10 and 5 Combinations of 3 and 4 Components of Metabolic Syndrome, Respectively						
Variable	Odds ratio of chronic kidney disease (95% CI)					
	UnadjustedOR (95% CI)	p-value^b^	Age-gender- adjustedOR (95% CI)	p-value^b^	Multivariable adjusted^a^OR (95% CI)	p-value^b^
No components	Reference		Reference		Reference	
EBP+AO+LHDL (n = 947)	9.59 (5.12–17.95)	<0.001	6.47 (3.38–12.41)	<0.001	6.11 (2.41–15.50)	<0.001
EBP+AO+TRG (n = 457)	13.90 (7.28–26.56)	<0.001	9.67 (4.83–19.37)	<0.001	10.32 (3.51–30.31)	<0.001
EBP+AO+FBG (n = 1130)	14.61 (7.85–27.19)	<0.001	10.09 (5.24–19.43)	<0.001	13.26 (5.21–33.72)	<0.001
EBP+LHDL+TRG (n = 343)	10.57 (5.43–20.56)	<0.001	7.31 (3.56–15.05)	<0.001	6.02 (2.02–17.89)	0.001
EBP+LHDL+FBG (n = 514)	11.62 (6.09–22.18)	<0.001	8.51 (4.22–17.15)	<0.001	9.44 (3.35–26.57)	<0.001
EBP+TRG+FBG (n = 290)	18.21 (9.34–35.53)	<0.001	14.13 (6.66–29.97)	<0.001	14.98 (4.59–48.92)	<0.001
AO+LHDL+TRG (n = 337)	10.14 (5.21–19.74)	<0.001	6.84 (3.39–13.80)	<0.001	5.09 (1.65–15.73)	0.005
AO+LHDL+FBG (n = 543)	11.03 (5.78–21.03)	<0.001	8.70 (4.34–17.45)	<0.001	10.01 (3.46–28.96)	<0.001
AO+TRG+FBG (n = 276)	19.55 (9.99–38.27)	<0.001	16.59 (7.82–35.19)	<0.001	25.11 (6.94–90.90)	<0.001
LHDL+TRG+FBG (n = 202)	13.85 (6.89–27.85)	<0.001	11.92 (5.28–26.89)	<0.001	13.92 (3.40–56.98)	<0.001
EBP+AO+LHDL+TRG (n = 280)	12.11 (6.16–23.81)	<0.001	8.28 (3.97–17.25)	<0.001	6.71 (2.01–22.33)	0.002
EBP+LHDL+TRG+FBG (n = 183)	14.37 (7.08–29.16)	<0.001	11.69 (5.11–26.76)	<0.001	15.01 (3.58–62.90)	<0.001
EBP+AO+TRG+FBG (n = 249)	21.31 (10.81–42.02)	<0.001	16.43 (7.64–35.32)	<0.001	27.06 (7.21–101.60)	<0.001
BP+AO+HDL+FBG (n = 456)	11.96 (6.23–22.84)	<0.001	9.08 (4.47–18.46)	<0.001	11.68 (3.93–34.70)	<0.001
AO+LHDL+TRG+FBG (n = 178)	15.03 97.38–30.63)	<0.001	12.87 (5.61–29.52)	<0.001	18.86 (3.89–91.73)	<0.001

OR, odds ratio; CI, confidence interval; HDL, high density lipoprotein.

AO, abdominal obesity; TRG, elevated triglycerides; EBP, elevated blood pressure; FBG, elevated fasting glucose; LHDL, Low high-density lipoprotein.

a Adjusted for age, gender, education, income, active living index, body mass index (BMI), cigarette smoking, and non-steroidal anti-inflammatory drugs (NSAIDs).

b P-values calculated using logistic regression.

abdominal obesity, defined by a waist circumference ≥102 cm in men and ≥88 cm in women; elevated blood pressure, defined as a systolic blood pressure of 130 mm Hg or greater and/or a diastolic blood pressure of 85 mm Hg or greater, physician diagnosis, and/or use of antihypertensive drugs; elevated plasma triglycerides (≥150 mg/dL) or treated dyslipidemia; low fasting HDL cholesterol (men <40 mg/dL and women <50 mg/dL; and elevated fasting glucose (≥100 mg/dL) or use of anti-diabetic medication.

Metabolic syndrome defined as having three or more of the following: abdominal obesity, elevated blood pressure, fasting blood glucose, triglycerides and low HDL cholesterol.

## Discussion

To our knowledge, this investigation is the first to examine the associations between individual MetS components, the presence of MetS, the number of MetS components and various combinations of MetS components and CKD among AAs. The findings reveal significant relationships between MetS, the presence of individual MetS components, the number of MetS components and certain combinations of MetS components, and CKD independent of age, gender, education, income, active living index, BMI, cigarette smoking, and NSAIDs. These findings are noteworthy because ten combinations of three MetS components and five combinations of four components identified are associated with the risk of CKD among AAs. The findings have important clinical and public health implications for three reasons: 1) the prevalence of components of MetS (elevated blood pressure, elevated glucose, and abdominal obesity) is high in AA populations [18], [26], 2) AAs have a disproportionately higher burden of CKD than white Americans [27], and 3) the state of Mississippi has a very high proportion (37%) of AAs in its population. In addition, MetS is linked with an increased risk of morbidity and mortality for CVD [28], which is the leading cause of death in Mississippi with death rates disproportionately higher for AAs than whites and Mississippi has among the highest CVD death rate nationally [29]. Finally, this investigation contributes to the scarce literature documenting the relationship between MetS and CKD among AAs, a population whose risk of CKD progressing to ESRD is higher than other ethnic groups [7].

Using the ATP III definition for MetS [11], multivariable models demonstrated that both MetS and individual components of MetS, with the exception of low HDL cholesterol are independently associated with increased odds of having CKD in AA adults. This finding is consistent with a previous investigation of Korean adults [30] using the ATP III definition. The current results suggest that among AA adults in Mississippi, MetS significantly increased the odds of having CKD. The high prevalence of MetS components, namely elevated blood pressure and elevated fasting glucose, observed in this population may help explain these associations. The nonsignificant association between low HDL cholesterol and CKD may be partly due to the nonsignificant difference in mean fasting low HDL cholesterol between those with CKD compared to those without CKD. In addition, the low prevalence of the component relative to other components in this population may also contribute to the non-significance rather than the absence of an association.

Among JHS participants there was a significant positive association and linear trend between the number of MetS components and the prevalence of CKD, a finding that is consistent with other cross-sectional studies [12], [14], [30]. This finding suggests that for AA adults the number of MetS components present is strongly related to the presence of CKD. In addition, there was a significant positive linear relationship between the number of MetS components and the odds of CKD, which is also consistent with previous cross-sectional studies [14], [30]. The increased risk of CKD associated with a higher number of MetS components among AA adults highlights the potential additive detrimental impact [15]. In this study of AA adults there was not only a high prevalence of individual components of MetS, but also a high clustering of components, which could place this population at a higher risk of CKD.

Among the possible combinations of three MetS components, the combination of abdominal obesity, elevated triglycerides, and elevated fasting glucose was associated with the highest odds of CKD. Among the possible combinations of four MetS components, the combination of elevated blood pressure, elevated fasting glucose, elevated triglycerides and abdominal obesity was associated with the highest odds of CKD. Notably, abdominal obesity, elevated fasting glucose, and elevated triglycerides were in both of the combinations that had the highest odds for CKD. Of the combinations of three components, the three groups with the highest odds for CKD all included elevated fasting glucose. Similarly, of the combinations of four components, elevated fasting glucose was in three with the highest odds of CKD. Thus, in the focal population combinations of elevated fasting glucose and other MetS components seem to have an additive impact on the odds of CKD. This pattern highlights the impact of diabetes on the risk of CKD among AA adults. The significant relationships between certain combinations of MetS components and CKD observed in this population suggest the relative importance of these specific combinations. The relatively high prevalence of key MetS components in this population may partially explain these associations. Further studies are needed to explore this relationship, particularly among AAs.

One of the objectives of the Healthy People 2020 report is to reduce the proportion of the U.S. adult population with CKD by: 1) reducing the proportion of persons with CKD who have elevated lipids levels and blood pressure, and 2) increasing the proportion of persons with diabetes and CKD who receive medical evaluations [31]. In addition, the National Kidney Foundation Kidney Disease Outcomes Quality Initiative (NKF KDOQI) guidelines recommend screening individuals with diabetes and hypertension for undetected kidney disease during regular clinical visits because studies have shown that in individuals with diabetes and hypertension, keeping blood sugar and blood pressure under control prevented or delayed the onset of kidney disease [25], [32]. Identifying individuals with CKD in its initial stage and informing them of their condition will help these individuals live a healthy lifestyle and may prevent the progression to ESRD. However, awareness of CKD among JHS participants is reportedly low [3]. Similarly, among this population the treatment of CKD was fairly low (52%) compared to other chronic diseases such as such as hypertension (83.2%) and diabetes (85.4%) [3]. Shuval and colleagues (2011) [33] demonstrated that among AAs, vigorous physical activity was associated with the absence of MetS; this relationship is of great importance given the high level of physical inactivity (36.0%) among Mississippi adults and the high prevalence of MetS observed in among JHS participants (42.0%). In the 2011 Mississippi Behavior Risk Factor Surveillance System (BRFSS), AA male adults were more likely to smoke (33.3%) than white male adults (28.9%) (34) in addition, compared to white adult Mississippians, AAs adults were more likely to report having hypertension and diabetes, were less likely to have their cholesterol checked, and reported higher rates of obesity [34]. These disproportionate rates of poor health behaviors and outcomes among AA adults may explain the high prevalence of MetS and CKD observed in the JHS.

The analyses in this investigation revealed significant relationships between individual, combinations of MetS components and CKD as well as the overall presence of MetS in a high-risk population. Focused screening of AA adults using key MetS components may help prevent the onset of CKD, increase awareness of the condition, and alleviate the burden and associated healthcare costs in this high-risk population. Understanding the way in which specific combinations of MetS components are associated with the risk of CKD will facilitate the creation of focused interventions, treatments, and evidenced-based CKD screening strategies, especially among high-risk populations.

This study has many strengths including: use of a large, heterogeneous sample of AAs; standard phenotypically characterization; and more highly educated than participants in statistical metropolitan areas with similar multi-ethnic studies of CVD, thus making this sample more comparable to similar European American populations in similar metropolitan areas. However, it also has several potential limitations. First, because the analyses use cross-sectional data, causal relationships between MetS and CKD cannot be inferred from the results. Second, the focal cohort is AA adults and thus the findings are limited to this adult population [35]. Third, in this cohort GFR was not measured directly, and eGFR were calculated using the MDRD formula, which may not provide an accurate estimation of GFR in AA populations. In addition, the equation was developed among diseased patients and has not been validated in a general population, although the formula is used extensively in clinical practice for the assessment of renal injury [3], [30]. Lastly, the JHS sample was limited to non-institutionalized adults and was not designed to be a nationally representative sample. Given the sampling frame of the JHS cohort, findings from the study might not be generalized to all AAs.

We report that MetS is common in a heterogeneous population-based cohort of AA adults. Individual components of MetS, the number of MetS components and specific combinations of MetS components are independently associated with increased odds of CKD. Key specific combinations of MetS components were associated with higher odds of CKD, and the risk of CKD increased progressively as more MetS components were present. These current findings highlight the need for targeted prevention, screening, health promotion, and treatment intervention efforts related to MetS components among AA adults; a focus on the MetS components associated with the greatest increases in the risk of CKD should be a significant part of these efforts. Additional studies focused on AAs or other racial/ethnic groups should further investigate if treatment of the different combinations of MetS components modifies the risk of the development and progression of CKD.
